# Latitudinal differences on the global epidemiology of infantile spasms: systematic review and meta-analysis

**DOI:** 10.1186/s13023-018-0952-x

**Published:** 2018-11-29

**Authors:** Jason L. Jia, Shiyi Chen, Vishalini Sivarajah, Derek Stephens, Miguel A. Cortez

**Affiliations:** 1 Department of Medicine, University of Toronto, 190 Elizabeth Street R. Fraser Elliott Wing, Toronto, M5G 2C4 Canada; 2 Child Health Evaluative Sciences Research Program, SickKids Research Institute, The Hospital for Sick Children, Toronto, Canada; 3 Department of Pediatrics, Division of Neurology, University of Toronto, Toronto, Canada; 4 Neurosciences & Mental Health Program, Peter Gilgan Centre for Research and Learning, SickKids Research Institute, The Hospital for Sick Children, 555 University Avenue, Toronto, ON M5G 1X8 Canada

**Keywords:** Infantile spasms, Epidemiology, Meta-analysis

## Abstract

**Background:**

Infantile spasms represent the catastrophic, age-specific seizure type associated with acute and long-term neurological morbidity. However, due to rarity and heterogenous determination, there is persistent uncertainty of its pathophysiological and epidemiological characteristics. The purpose of the current study was to address a historically suspected latitudinal basis of infantile spasms incidence, and to interrogate a geographical basis of epidemiology, including the roles of latitude and other environmental factors, using meta-analytic and -regression methods.

**Methods:**

A systematic search was performed in Ovid MEDLINE and Embase for primary reports on infantile spasms incidence and prevalence epidemiology.

**Results:**

One thousand fifteen studies were screened to yield 54 eligible publications, from which 39 incidence figures and 18 prevalence figures were extracted. The pooled incidence was 0.249 cases/1000 live births. The pooled prevalence was 0.015 cases/1000 population. Univariate meta-regression determined a continental effect, with Europe demonstrating the highest onset compared from Asia (OR = 0.51, *p* = 0.004) and from North America (OR = 0.50, *p* = 0.004). Latitude was also positively correlated with incidence globally (OR = 1.02, *p* < 0.001). Sub-analyses determined a particularly elevated Scandinavian incidence compared to the rest of world (OR = 1.88, *p* < 0.001), and lack of latitudinal effect with Scandinavian exclusion (*p* = 0.10). Metrics of healthcare quality did not predict incidence. Multiple meta-regression determined that latitude was the key predictor of incidence (OR = 1.02, *p* = 0.001).

**Conclusions:**

This is the first systematic epidemiological study of infantile spasms. Limitations included lack of Southern hemispheric representation, insufficient study selection and size to support some sub-continental analyses, and lack of accessible ethnic and healthcare quality data. Meta-analyses determined a novel, true geographical difference in incidence which is consistent with a latitudinal and/or ethnic contribution to epileptogenesis. These findings justify the establishment of a global registry of infantile spasms epidemiology to promote future systematic studies, clarify risk factors, and expand understanding of the pathophysiology.

**Electronic supplementary material:**

The online version of this article (10.1186/s13023-018-0952-x) contains supplementary material, which is available to authorized users.

## Background

Infantile spasms (IS), the seizure type consisting of sudden truncal and/or limb flexion and/or extension occurring during the first year of life, are concerning for cognitive developmental stagnation and chronic neurological morbidity [1–4]. They therefore represent the eminent, catastrophic epileptic manifestation of infancy. Delay in diagnosis and treatment is a potentially modifiable risk factor [5], however identification of the factors leading to seizure onset has been hindered by the rarity and etiological heterogeneity of this seizure type. The age-specific incidence ranges from 0.2–0.5 cases/1000 live births (LB) and is associated with hundreds of abnormalities including brain malformations, hypoxic-ischemic encephalopathy, and genetic factors [2, 3].

Investigations of the epidemiology of IS have augmented the sparse knowledge of the seizure type. In 1991, Cowan & Hudson published the first, seminal review of global epidemiology, which included the finding of a 2.6-Fold increase of incidence in Finland compared to that of the U.S.A. [6]. This was preliminary evidence of a true geographical difference in IS onset, and consistent with the existence of an environmental risk to seizure development. However, there was insufficient primary data at that time to explore this hypothesis. In the ensuing 25 years, the geographical scope of the epidemiological database has expanded considerably without an updated review of environmental risk factors. The objective of the current study was to fulfil this knowledge gap: to systematically identify relevant reports, and establish the relationship between latitude and other geographical, temporal, and socioeconomic factors, on the incidence and prevalence of IS with meta-statistical methods.

## Methods

The aim of the study was to collect all epidemiological reports of IS up to 2016 from which we would determine summary incidence and prevalence and covariate associations using meta-analytic and meta-regression techniques. Electrographic correlation with hypsarrhythmia, the chaotic interictal pattern that, while associated with IS is not a defining feature of this seizure type, was not required [7]. Neurodevelopmental regression was not required because it was described in only few studies. IS was defined as the clinical presentation of sudden truncal or limb flexion, extension, or mixed flexion-extension lasting less than a few seconds. This seizure type is currently referred in the 2017 International League Against Epilepsy (ILAE) Classification of Seizures as epileptic spasms, a definition interchangeable with infantile spasms when present during the infantile period [8]. Distinction exists from other seizure types associated with early childhood, notably myoclonic which consist of shorter, irregular jerks, and tonic which demonstrate extended and sustained stiffening. Only studies explicitly reporting infantile spasms as a feature of West Syndrome (WS) [2], or describing seizures of truncal and/or limb extension and/or flexion of few seconds duration or less, and not consistent with other seizure types, were appropriate for meta-analysis inclusion. Confirmation of a study’s compatibility with this definition was made during study screening by two independent reviewers, as described below. Incidence referred to the number of diagnoses during observation. Prevalence referred to the number of patients with active symptoms during observation.

**Fig. 1:**
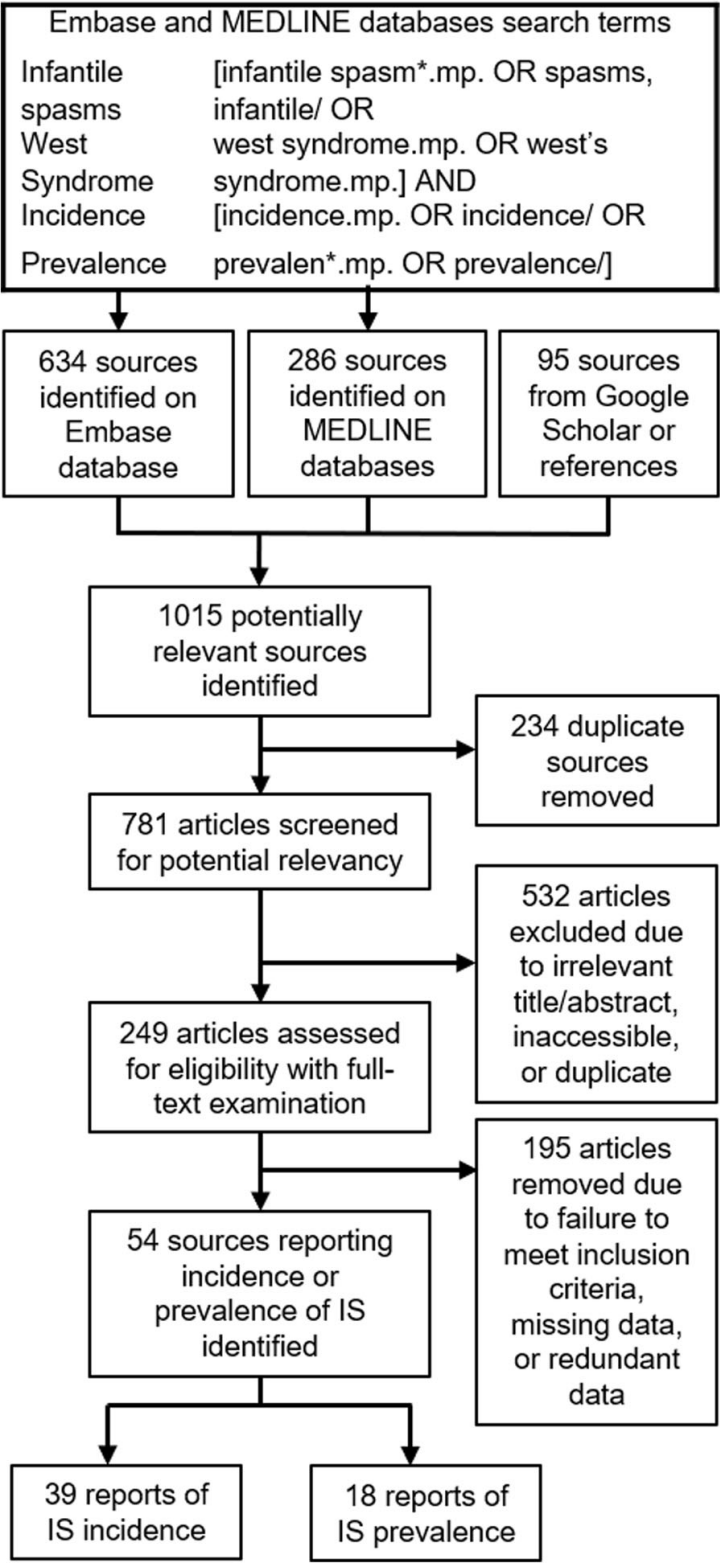
Search strategy and flow chart of study selection. MeSH and free-text entries of IS/WS and epidemiological outcomes were applied to Medline and EMBASE databases for screening. In total, 1015 articles were identified during the primary search, and then systematically assessed for relevance to the research question. 54 articles were included [15–68], 3 of which reported both the incidence and prevalence of IS [15, 19, 30]. Thus, 57 data points were abstracted: 39 incidence figures. [15–53] and 18 prevalence figures. [15, 19, 30, 54–68]

This systematic review and meta-analysis was conducted in accordance to Preferred Reporting Items for Systematic Reviews and Meta-Analyses guidelines [9]. WS (triad of IS, hypsarrhythmia and abnormal cognition) was incorporated in the search for studies on IS, as this epileptic syndrome definitionally requires presence of IS, in addition to hypsarrhythmia. The search was conducted on MEDLINE (1946–2016) and Embase (1947–2016) using MeSH and free-text entries of “West Syndrome” and “infantile spasms” (Fig. 1). It encompassed all publications plus conference abstracts published until July 11, 2016. There was no language restriction for the inclusion of primary incidence and prevalence figures. Additional searches were conducted on Google Scholar and the University of Toronto libraries database. Studies were catalogued in Excel 2016.

Two independent reviewers (JLJ & VS) determined study eligibility. First-stage screening excluded all publications with titles or abstracts irrelevant to pediatric epilepsy epidemiology. During second-stage screening, publications were admitted into the epidemiological database based on full-text assessment and if the study reported the incidence or prevalence of IS patients, number of cases, sample size, and target population age range. Inability to fill missing data, despite exhaustive literature/public database searches and direct correspondence with study authors, warranted exclusion. Conflicts of study inclusion were reconciled between reviewers. Eligible studies were classified as incidence, prevalence, or both.

The two reviewers manually abstracted target catchment area, ascertainment period, number of cases and corresponding sex ratio, age-of-onset of IS, number of live births, target age-range, total catchment population, catchment or regional sex ratio, and incidence/age-specific prevalence from the eligible studies. Published prevalence figures were determined based on unequal lengths of follow-up which prohibited between-study comparability due to the age-specific nature of IS. To overcome this obstacle, these available prevalence figures were age-standardized by multiplying the age-specific prevalence by the proportion of the age-specific population within the total population, a method described in the Centers for Disease Control statistical manual [10]. Latitude of study centers were identified with the NASA resource “Latitude/Longitude Finder” (https://www.latlong.net/). The geographical midpoint was selected for expansive catchment areas. Time was calculated as the midpoint of the ascertainment period (midpoint year), or as the prevalence point. Gross Domestic Product (GDP) per capita of the midpoint year was recorded based on government report and inflation-adjusted to 2016 United States Dollars (USD). Measures of national healthcare quality included physicians per capita, health expenditure per capita, and health expenditure as percentage of GDP. These figures were abstracted for each of the incidence countries from the World Health Organization Global Health Repository from 2014, 2015, or 2016 as the data was rarely available for midpoint years [11]. National ethnic distributions for the incidence countries were also drawn from the CIA World Factbook [12] and converted to a binary variable according to a 50% Caucasian proportion threshold. Datasets of the incidence and prevalence databases, and the national healthcare/ethnicity metrics, are available in the Additional files 1, 2 and 3 section. Each publication was independently evaluated for methodological quality using a 7 point system modified from that of Loney et al. [13]. The following 7 factors were assessed: appropriate study design, normal sampling frame, adequate sample size, standard ascertainment criteria, unbiased diagnosticians, high response rate, and sufficient data published. We initially determined a sample size of 65,900 live births (LB) required to detect IS incidence from 0.16 and 0.42 cases/1000 LB with 95% confidence. 7,516,000 population was necessary to detect prevalence in the range of 0.018 to 0.025 cases/1000 with 95% confidence. Scores of 1–2, 3–4, and 5–7 represented low, medium, and high quality respectively.

Comprehensive Meta-Analysis version 3.0 software was used for statistical analyses and figure creation [14]. Effect sizes represented cumulative incidence or prevalence and were indexed as event rate. Incidence was expressed as the number of new cases/1000 LB and prevalence was expressed as the number of active cases/1000 population. Two meta-analyses were conducted for incidence and prevalence. Forest plots presented the range of outcome data and the summary effect sizes as calculated with the random-effects model and inverse-variance weighting. Heterogeneity of between-study variance was assessed using Q test of homogeneity and I^2^ statistics. I^2^ statistics of 25, 50 and 75% represented low, medium and high heterogeneity respectively. Funnel plots with Duval and Tweedie’s Trim and Fill method assessed publication bias. For incidence studies, univariate and multiple meta-regression models were constructed to determine associations between incidence and covariates including continent, latitude, time, and GDP per capita. Effect sizes were expressed as odds ratios (OR) with accompanying R^2^ analogs. Random-effects modeling was appropriate due to its conservative approach to within-study sampling error and between-study variance. The same procedure was repeated with prevalence studies. Additional meta-regression models determined associations between latitude and age-of-onset of IS, and between-study sex ratio and regional population sex ratio.

## Results

### Study selection

One thousand fifteen articles were identified in the primary searches. 234 duplicate sources were removed. 532 were excluded during first-stage screening. Of the remaining 249 studies which underwent full-text assessment, 195 were removed due to incompatibility with inclusion criteria or redundant data. The remaining 54 studies encompassed 22 countries in the Northern hemisphere [15–68], 13 of which were located in the Scandinavian nations of Denmark, Sweden, Finland, and Iceland. There was no identification of studies from the Southern hemisphere. 3 studies reported both the incidence and prevalence of IS [15, 19, 30]. Thus, 57 data points were abstracted: 39 incidence figures. [15–53] and 18 prevalence figures. [15, 19, 30, 54–68] (Fig. 1). Incidence, midpoint, and unadjusted and age-adjusted prevalence figures were extracted (Table 1). Periods of observation amongst incidence and prevalence studies ranged from 1957 to 2014 and 1975 to 2009 respectively. The incidence database consisted of 22 European, 9 Asian, and 8 North American publications (Fig. 2), and the prevalence database consisted of 10 European, 5 Asian, and 3 North American publications (Fig. 3) [69]. 5 studies reported the age-of-onset of IS [28, 34, 37, 44, 48], 14 incidence [17, 20, 22, 27–29, 31, 34, 35, 37, 38, 44, 48, 51], and 4 prevalence [15, 54, 58, 64] studies reported sexual distribution.

**Table 1:** Summary of studies reporting the incidence and/or prevalence of IS

	INC or PRV	Date	Country	Latitude (^o^N)	Cases	Event Rate	Age-Adjusted
Bobo et al. (1994) [15]	INC	1987–1988	U.S.A.	45.55	11	0.100	
Braathen & Theorell (1995) [16]	INC	1990–1992	Sweden	59.24	1	0.096	
Brna et al. (2001) [17]	INC	1978–1998	Canada	46.34	75	0.307	
Camfield et al. (1996) [18]	INC	1977–1985	Canada	44.68	32	0.323	
Chen et al. (2004) [19]	INC	1985–1997	Taiwan	25.03	2	0.060	
Cortez et al. (1997) [20]	INC	1984–1993	Canada	43.65	76	0.056	
Doerfer & Wasser (1987) [21]	INC	1982–1985	Germany	48.55	3	0.570	
Doose & Sitepu (1982) [22]	INC	1957–1966	Germany	54.32	18	0.478	
Dura-Trave et al. (2008) [23]	INC	2002–2005	Spain	42.70	10	0.301	
Eltze et al. (2013) [24]	INC	2005–2006	U.K.	51.51	16	0.300	
Freitag et al. (2001) [25]	INC	1999–2000	Germany	49.45	0	0.000	
Heijbel et al. (1975) [26]	INC	1973–1974	Sweden	65.33	1	0.317	
Hino-Fukuyo et al. (2009) [27]	INC	2000–2005	Japan	38.27	45	0.420	
Howitz & Platz (1978) [28]	INC	1976–1977	Denmark	56.26	21	0.322	
Hwang (2001) [29]	INC	1997–2000	South Korea	35.91	324	0.168	
Joensen (1986) [30]	INC	1970–1980	Denmark	61.89	4	0.467	
Lee & Ong (2001) [31]	INC	1998–1999	Singapore	1.35	9	0.310	
Loiseau et al. (1990) [32]	INC	1984–1985	France	44.85	8	0.535	
Lommi et al. (2010) [33]	INC	1997–2006	Finland	60.17	48	0.376	
Ludvigsson et al. (1994) [34]	INC	1981–1990	Iceland	64.96	13	0.302	
Matsuo et al. (2001) [35]	INC	1989–1998	Japan	32.74	47	0.310	
Olafsson et al. (2005) [36]	INC	1995–1999	Iceland	64.96	6	0.474	
Primec et al. (2002) [37]	INC	1985–1995	Slovenia	46.15	47	0.206	
Rantala & Putkonen (1999) [38]	INC	1976–1993	Finland	65.01	37	0.410	
Riikonen (1995) [39]	INC	1960–1991	Finland	60.22	209	0.425	
Saemundsen et al. (2007) [40]	INC	1981–1998	Iceland	64.96	25	0.338	
Sarsenbayeva et al. (2015) [41]	INC	2013–2014	Kazakhstan	48.02	60	0.154	
Schmitt et al. (1996) [42]	INC	1991–1991	Germany	51.26	4	0.180	
Shields et al. (1988) [43]	INC	1967, 1972	Denmark	56.26	80	0.513	
Sidenvall & Eeg-Olofsson (1995) [44]	INC	1987–1991	Sweden	61.38	57	0.450	
Trevathan et al. (1999) [45]	INC	1975–1977	U.S.A.	33.75	20	0.290	
Tsuboi (1988) [46]	INC	1974–1980	Japan	35.67	3	0.175	
Van Den Berg et al. (1969) [47]	INC	1960–1968	U.S.A.	37.80	3	0.162	
Vardi et al. (2005) [48]	INC	1981–1997	Israel	32.02	31	0.220	
Verity et al. (1992) [49]	INC	1970–1980	U.K.	55.38	3	0.187	
Wendt et al. (1985) [50]	INC	1966–1966	Finland	66.47	7	0.581	
Wirrell et al. (2011) [51]	INC	1980–2004	U.S.A.	44.00	9	0.198	
Young (2001) [52]	INC	1998–1999	Taiwan	23.70	41	0.074	
Zarrelli et al. (1999) [53]	INC	1980–1984	U.S.A.	44.01	0	0.000	
Beilmann et al. (1999) [54]	PRV	1997	Estonia	58.38	8	0.0138	0.0139
Bobo et al. (1994) [15]	PRV	1988	U.S.A.	45.55	21	0.0028	0.0028
Chen et al. (2004) [19]	PRV	1997	Taiwan	25.03	142	0.0065	0.0065
Cowan et al. (1989) [55]	PRV	1983	U.S.A.	35.01	23	0.0327	0.0328
Dura-Trave et al. (2012) [56]	PRV	2009	Spain	42.70	24	0.0396	0.0397
Endziniene et al. (1997) [57]	PRV	1995	Lithuania	54.90	3	0.0072	0.0066
Eriksson & Koivikko (1997) [58]	PRV	1992	Finland	61.50	25	0.0578	0.0580
Granieri et al. (1983) [59]	PRV	1978	Italy	44.89	1	0.0221	0.0222
Ishida (1985) [60]	PRV	1975	Japan	34.66	41	0.0225	0.0226
Joensen (1986) [30]	PRV	1980	Denmark	61.89	4	0.0917	0.0917
Koul et al. (1988) [61]	PRV	1986	India	33.70	4	0.0628	0.0628
Kurth et al. (2010) [62]	PRV	2006	U.S.A.	37.09	59	0.0042	0.0009
Kwong et al. (2001) [63]	PRV	1997	China	22.41	16	0.0142	0.0143
Larsson & Olofsson (2006) [64]	PRV	2000	Sweden	59.86	4	0.0135	0.0136
Maremmani et al. (1991) [65]	PRV	1985	Italy	43.78	0	0.0000	0.0000
Oka et al. (2001) [66]	PRV	1994	Japan	34.66	6	0.0031	0.0031
Olafsson & Hauser (1999) [67]	PRV	1993	Iceland	64.96	2	0.0223	0.0223
Sidenvall et al. (1996) [68]	PRV	1985	Sweden	65.33	3	0.0180	0.0181

39 incidence (INC) and 18 prevalence (PRV) data points were abstracted from 54 studies [15–68]. For incidence studies, date refers to the period of observation and event rate is the number of new cases per 1000 live births. For prevalence studies, date refers to the prevalence point, event rate is the age-specific prevalence, and age-adjustment lists the age-standardized figure per 1000 population

**Fig. 2:**
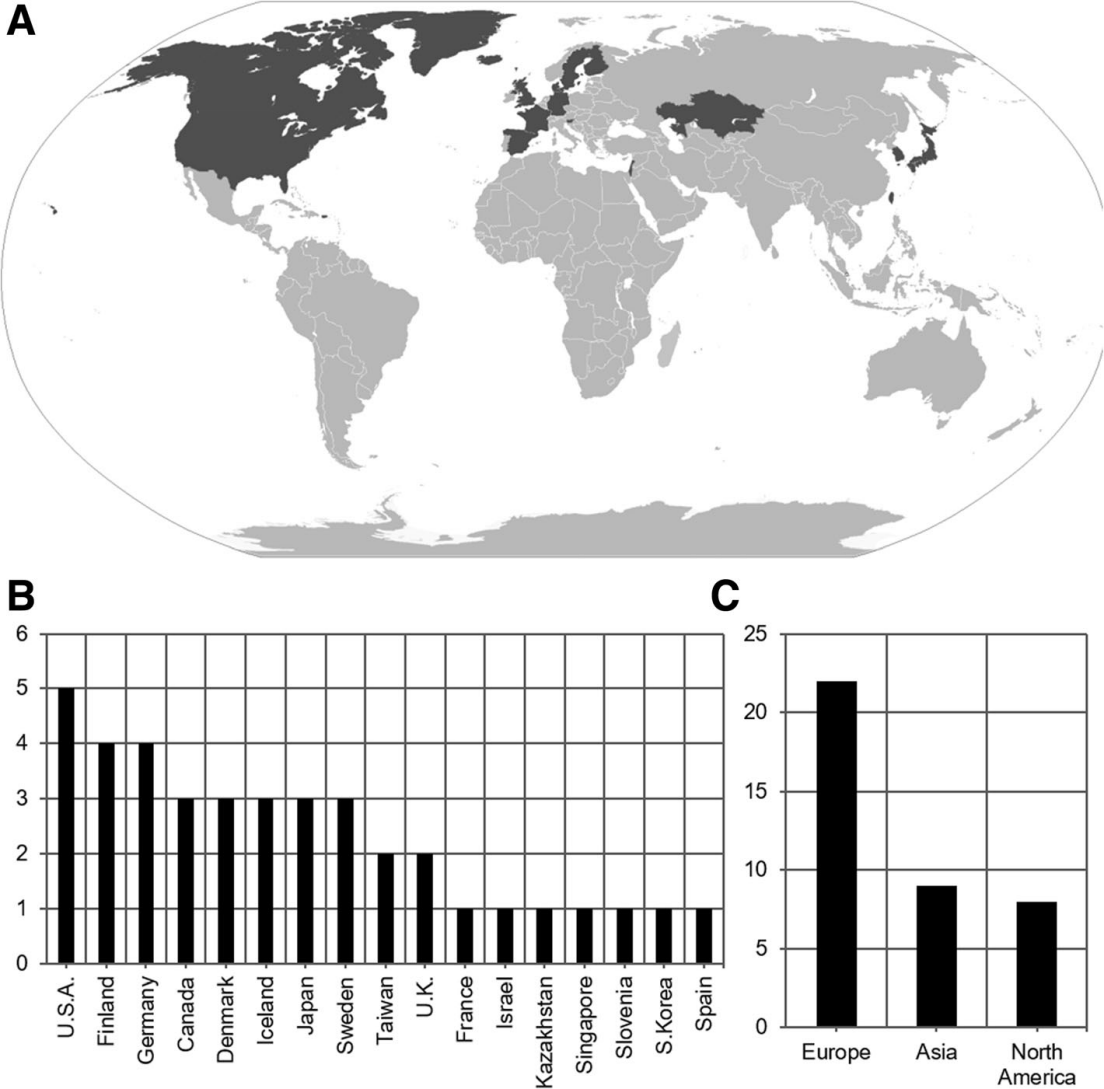
Geographical distribution of incidence studies. 39 studies [15–53] reported incidence globally. Varying tiers of regional observation with some cross-coverage were reported, from municipal to national levels. Study targets, including their administrative level if not specific, consisted of, from approximately West to East longitude: Oregon, Oakland, Ontario, Rochester, Olmsted County, Atlanta, Nova Scotia, Prince Edward Island, Iceland, Faroe Islands, United Kingdom, Navarre, Gironde, London, Germany, Heidelberg, Mannheim, Altenburg District, Denmark, Kiel, Slovenia, Uppsala, Vasterbotten County, Huddinge, Helsinki, Uusimaa County, Oulu, Lapland, south Israel, Kazakhstan, Singapore, Taiwan, Taipei, South Korea, Nagasaki Prefecture, Fuchu City, Miyagi Prefecture. **a** World map with national boundaries, adapted from [69]. Countries highlighted correspond to sites in which incidence studies were performed. **b** Bar chart of number of incidence studies published by country. **c** Bar chart of number of incidence studies published by continent. No studies were identified from South America, Africa, or Oceania

**Fig. 3:**
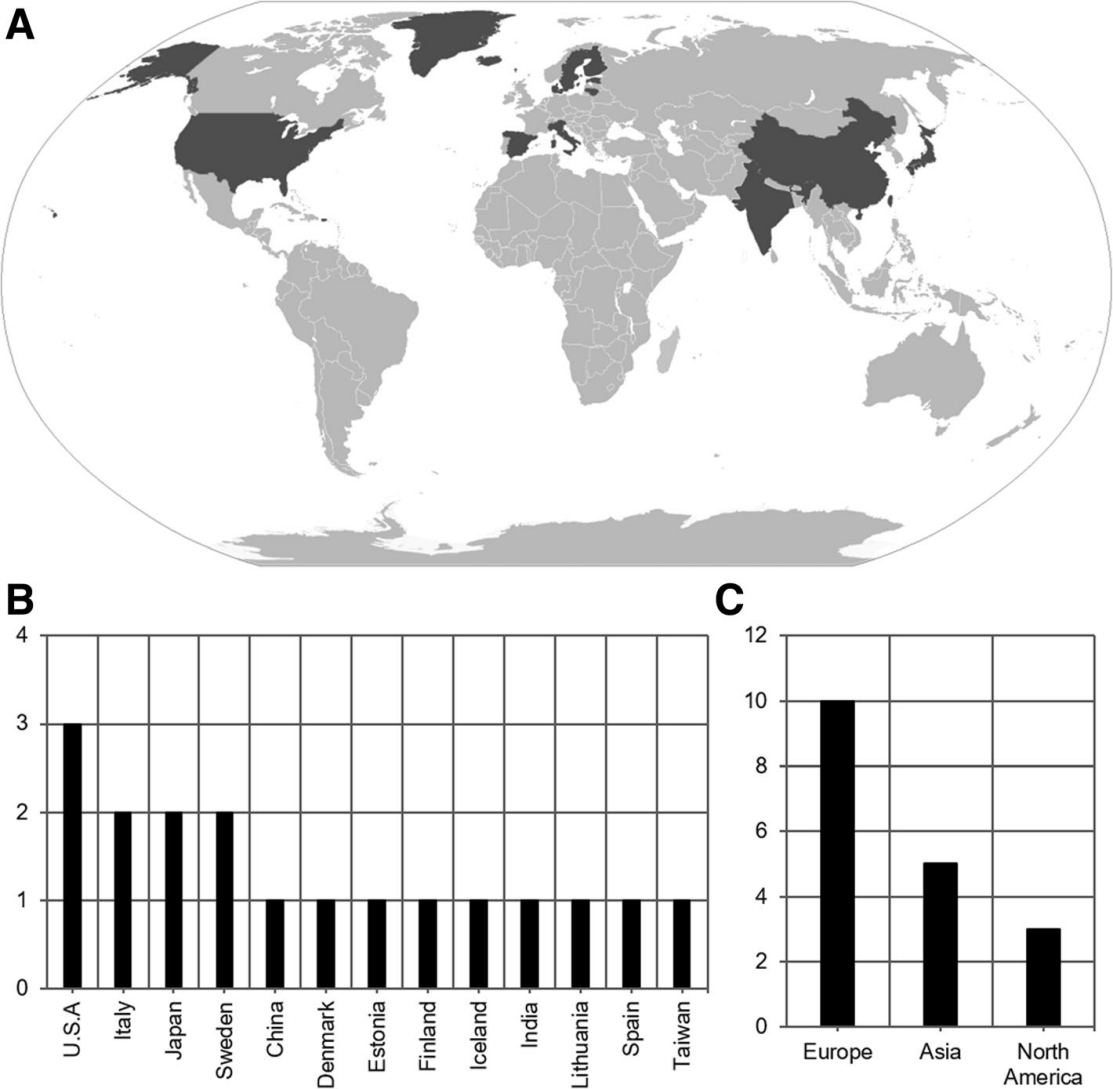
Geographical distribution of prevalence studies, 18 studies [15, 19, 30, 54–68] reported prevalence globally. Varying tiers of regional observation with some cross-coverage were reported, from municipal to national levels. Study targets, including their administrative level if not specific, consisted of, from approximately West to East longitude: Oregon, Washington State, Oklahoma, U.S.A, Iceland, Faroe Islands, Navarre, Vecchiano, Copparo, Vasterbotten County, Uppsala, Tampere, Kaunas, Tartu, Kuthar Valley, Hong Kong, Taipei, Okayama Prefecture. **a** World map with national boundaries, adapted from [69]. Countries highlighted correspond to sites in which prevalence studies were performed. **b** Bar chart of number of prevalence studies published by country. **c** Bar chart of number of prevalence published by continent. No studies were identified from South America, Africa, or Oceania

### Methodological assessments

Risk of bias assessment determined an average total score of 5.05 (SD = 1.47) out of 7 for the incidence database. 92% of studies in this database had appropriate study design, 46% had adequate sample size, 90% had a representative sampling frame, 74% had standard diagnostic criteria, 62% had objective case assessors, 87% had high response rate, and 54% reported sufficient data. The Scandinavian selection specifically had a total score of 4.71 compared to the non-Scandinavian (Equatorial) selection score of 5.04. Of these regions respectively, 86 and 92% had appropriate study design, 43 and 46% had adequate sample size, 93 and 85% had a representative sampling frame, 64 and 77% had standard diagnostic criteria, 57 and 62% had objective case assessors, 79 and 89% had high response rate, and 50 and 54% reported sufficient data.

The prevalence dataset had an average total score of 5.17 (SD = 0.62), of which 100% of studies had appropriate study design, 16% had adequate sample size, 90% had a representative sampling frame, 95% had standard diagnostic criteria, 32% had objective case assessors, 74% had high response rate, and 100% reported sufficient data.

### Meta-analyses: Summary incidence and prevalence

**Fig. 4:**
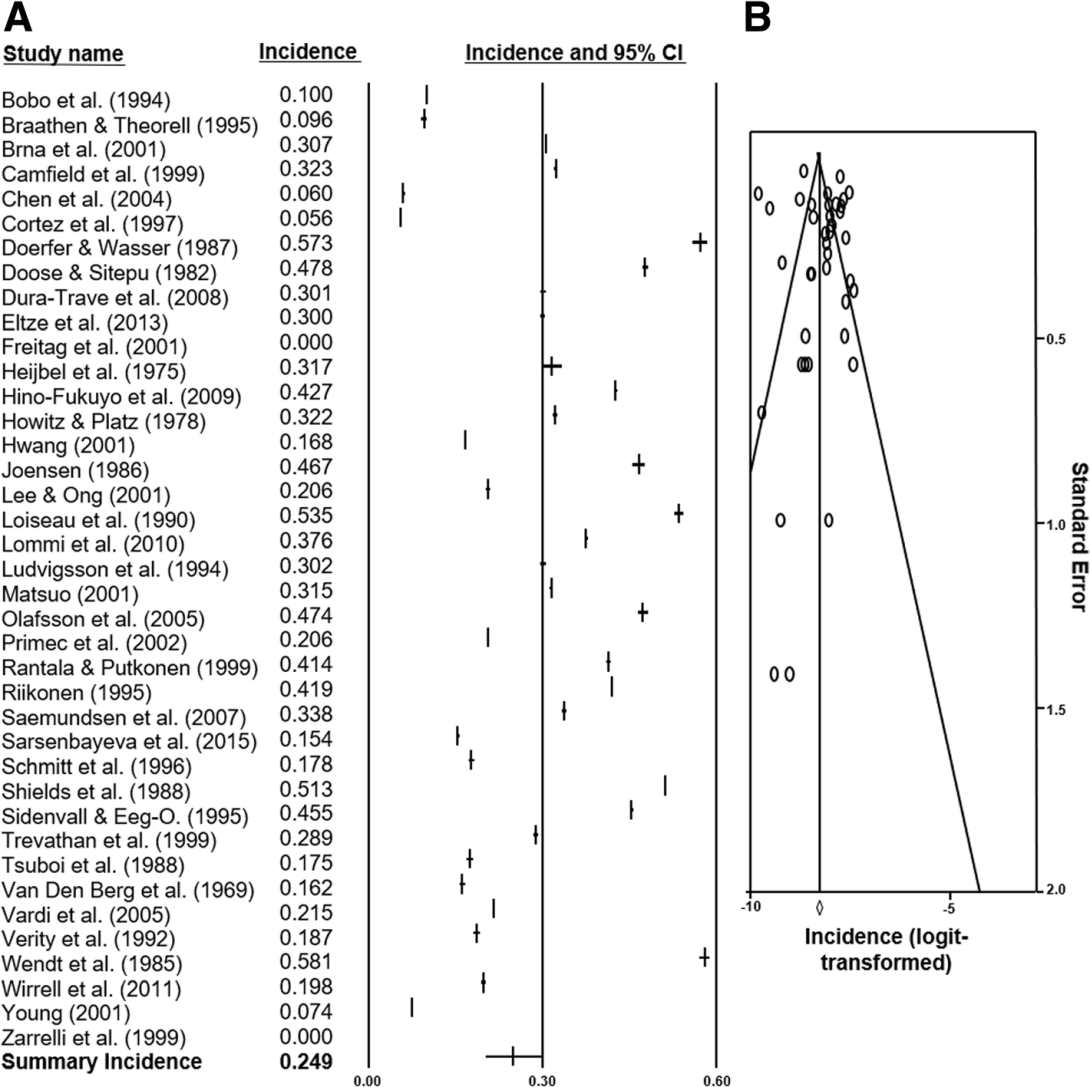
Forest plot and funnel plot of incidence studies included in analysis. **a** 39 studies [15–53] and their incidence statistics are listed individually. The summary rate, calculated with meta-analysis and random-effects modelling, is listed on the final row. **b** 39 logit-transformed incidence figures are plotted against their individual standard errors

**Fig. 5:**
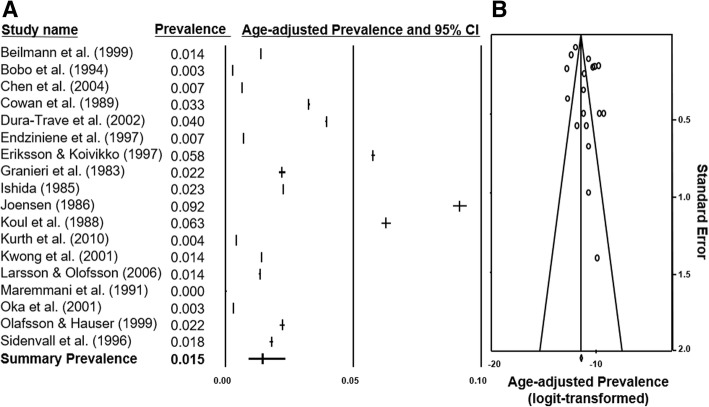
Forest plot and funnel plot of prevalence studies included in analysis. **a** 18 studies [15, 19, 30, 54–68] and their prevalence statistics are listed individually. The final row indicates the summary prevalence calculated with random-effects modelling. **b** 18 logit-transformed age-adjusted prevalence figures are plotted against their individual standard errors

Two meta-analyses of the incidence and prevalence study selections were constructed. From 39 incidence figures, a summary rate of 0.249 cases/1000 LB (95% CI 0.202–0.303) with Q statistics of 502.04 (*p* < 0.001) and I^2^ of 92.70 was determined. The summary prevalence from 18 data points was 0.015 cases/1000 population (95% CI 0.009–0.024) with Q statistics of 333.42 (*p* < 0.001) and I^2^ of 94.90. Forest-plots of the databases were constructed (Fig. 4a and Fig. 5a). The summary incidence of each country was also calculated by meta-analyses. Of note, the pooled incidence in Finland was 0.446/1000 LB (95% CI 0.408–0.485) and in the U.S.A. was 0.134/1000 LB (95% CI 0.076–0.225) which represented an approximate 3.3-Fold increase. The incidence in Europe was 0.358/1000 LB (95% CI 0.309–0.415), in Asia was 0.18/1000 LB (95% CI 0.121–0.268), and in North America was 0.17/1000 LB (95% CI 0.086–0.337).

### Univariate meta-regression: Latitude and continent

**Fig. 6:**
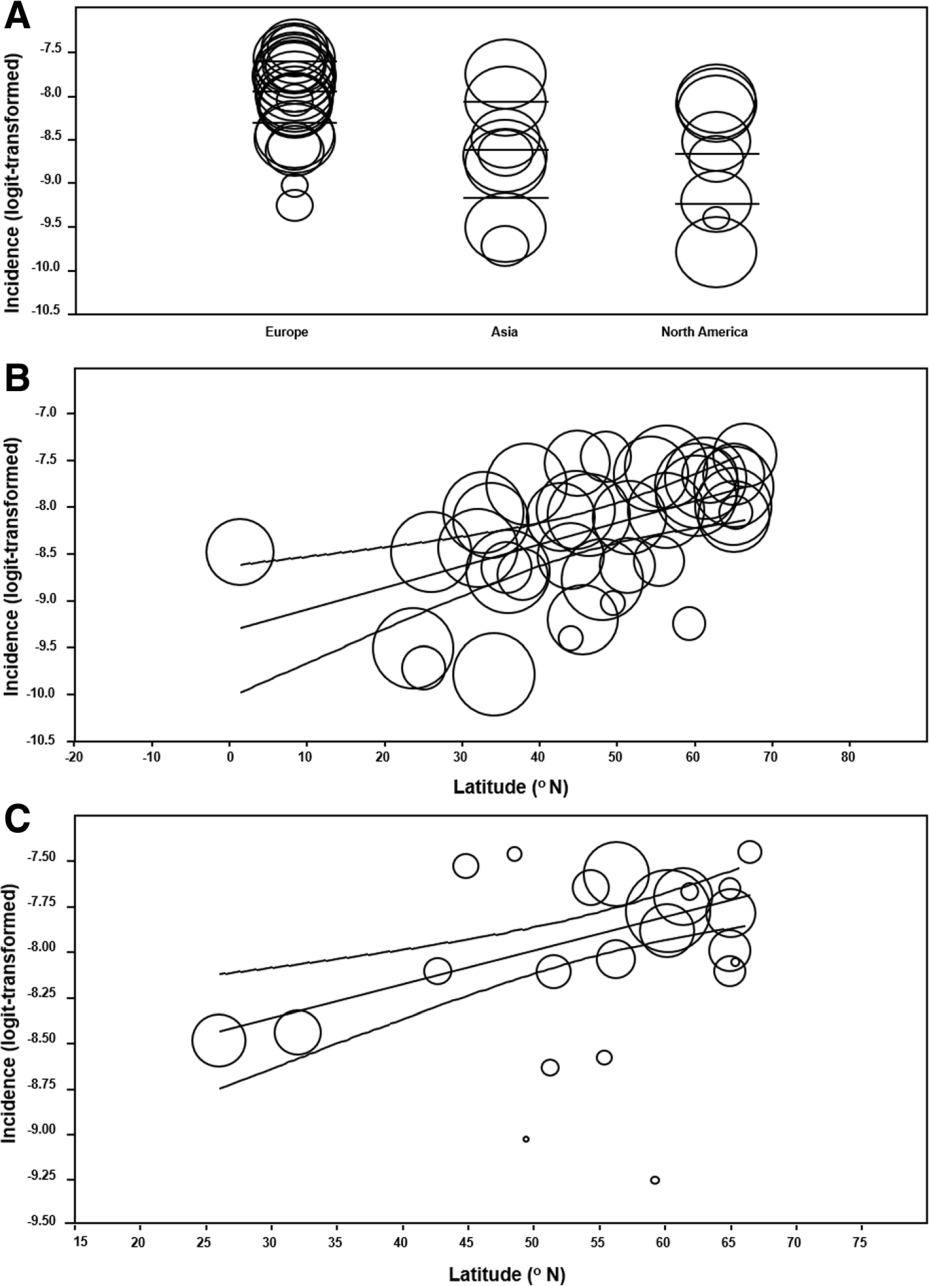
Scatter plots of incidence and geographical covariates. Incidence of infantile spasms abstracted from 39 studies [15–53] are represented as circles proportional to sample size and arranged in scatter plots according to continent and latitude. **a** Global incidence is categorized by continent with mean and 95% confidence intervals (CIs) also shown, consisting of 22 European studies, 9 Asian studies, and 8 North American reports. No South American, African, or Oceanic reports were identified. **b** Global incidence of IS is plotted against latitude. The lines indicate line of regression and associated 95% CIs. **c** Incidence within Europe only plotted against latitude. 95% CIs and line of regression are also shown

Meta-regression between continent and incidence was significant (R^2^ analog = 0.35; *p* = 0.0015) (Fig. 6a). Compared to European counterparts, Asian infants were 49% less likely to have IS onset (OR = 0.51; 95% CI 0.33–0.81, *p* = 0.0042) and North American infants were 50% less likely (OR = 0.50; 95% CI 0.31–0.80, *p* = 0.0037). The European sources encompassed a latitudinal range of 26.03^o^N to 66.47^o^N which is polar-centric compared to that of Asia (1.35^o^N to 48.02^o^N) and North America (33.75^o^N to 46.34^o^N). Meta-regression with studies only in the 23.70^o^N to 46.34^o^N range, excluding 18 outlying sites, showed no effect (*p* = 0.33). Identical analytical procedure with the prevalence database also showed no effect (*p* = 0.10).

The univariate meta-regression model of latitude and incidence globally determined a positive effect (OR = 1.02; 95% CI 1.01–1.04; R^2^ analog = 0.49; *p* < 0.001) (Fig. 6b). There was no association between latitude and prevalence (*p* = 0.26). Sub-continental meta-regression analyses showed no latitudinal effect upon incidence within North America (*p* = 0.42), and Asia (*p* = 0.58), yet a positive correlation was detected within Europe (OR = 1.02; 95% CI 1.01–1.03; R^2^ analog = 0.87, *p* < 0.001) (Fig. 6c).

**Fig. 7:**
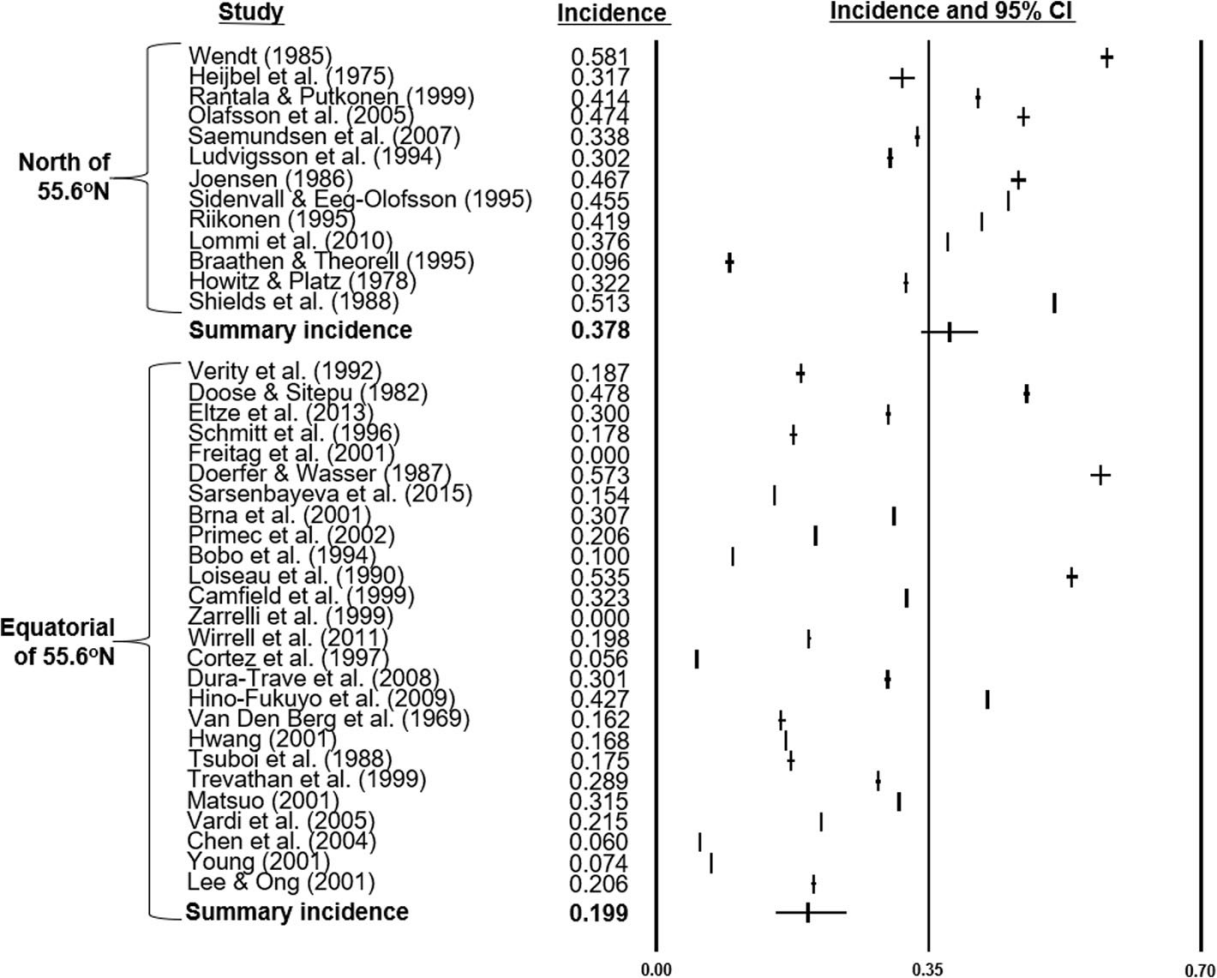
Forest plots of incidence studies above and below 55.6^o^N latitude. 39 studies [15–53] and their incidence statistics are listed individually and arranged in order of decreasing latitude, separated into 13 publications North of 55.6^o^N [16, 26, 28, 30, 33, 34, 36, 38–40, 43, 44, 50], which were exclusively Scandinavian, and 26 remaining Equatorial publications. Summary rates for the groups are noted

**Fig. 8:**
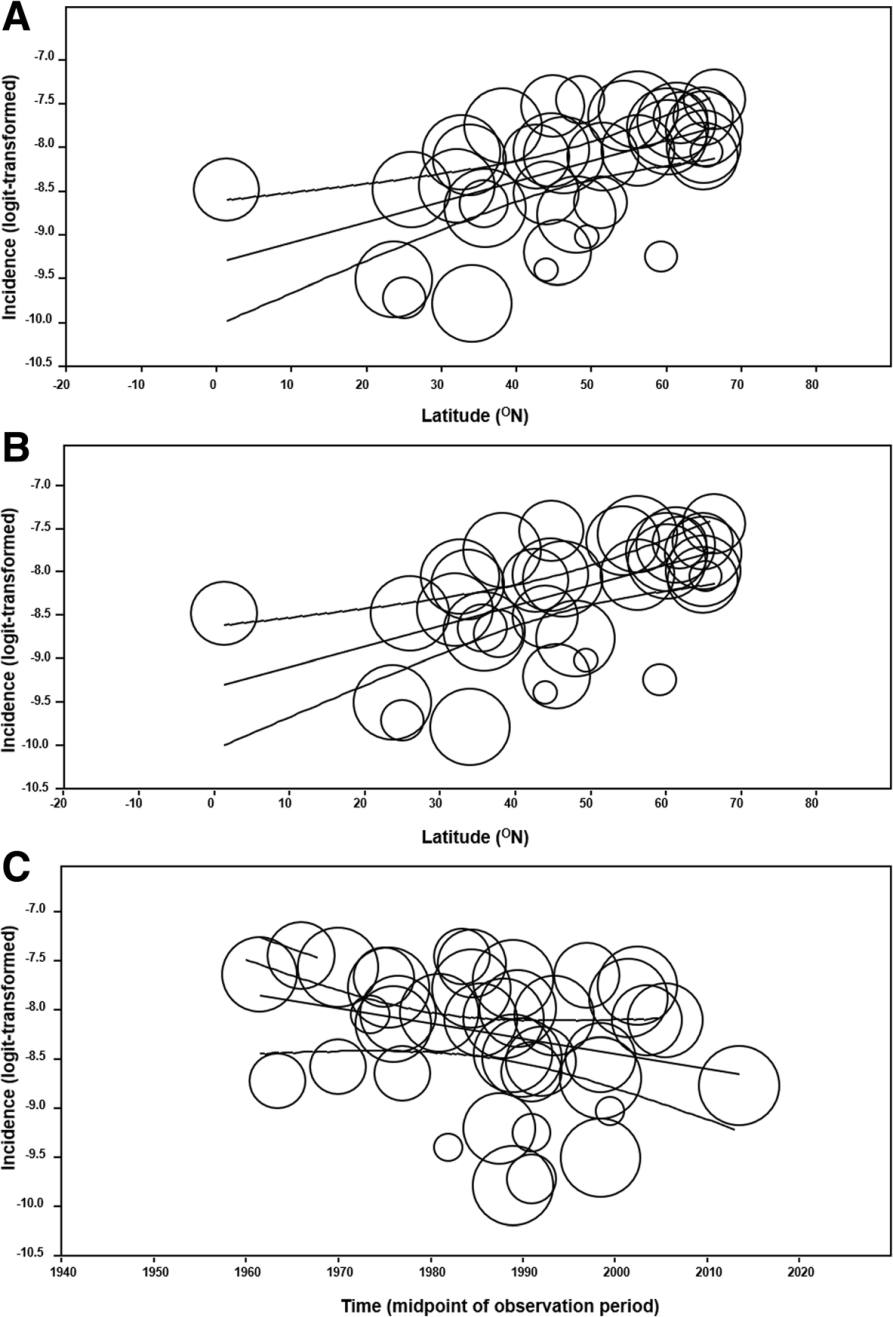
Additional scatterplots of incidence 39 global studies [15–53] reporting incidence, individually represented as circles with size proportional to sample size, are visualized. **a** 2 cohort studies [47, 49] were removed from the global latitudinal selection before re-plotting with remaining 37 figures. 95% confidence intervals (CIs) and line of regression are shown. **b** 4 community-based studies [21, 24, 42, 49] were removed from the global latitudinal selection before re-plotting with remaining 35 figures. 95% CIs and line of regression shown. **c** Global incidence plotted against time, defined here as the midpoint of observation period. Studies are represented as circles proportional to sample size. 95% CIs and line of regression are shown. The observation period of all incidence studies extended 1957 to 2014 with midpoints ranging from 1961 to 2013. Corresponding univariate meta-regression analysis demonstrated no effect of time on incidence globally (*p* = 0.07)

To clarify the European trend, countries North of the 55.65^o^N latitudinal threshold (*n* = 13), which were exclusively Scandinavian (Iceland, Finland, Sweden, Denmark), were analyzed separately. The regional summary incidence was 0.378 cases/1000 LB (SD = 0.342–0.416), without latitudinal effect (*p* = 0.28), and non-significant goodness-of-fit (Q = 10.26; df = 11; *p* = 0.51). The remaining 26 studies spanned 55.38^o^N to 1.35^o^N with summary incidence of 0.199 cases/1000 LB (SD = 0.156–0.250), no latitudinal effect (*p* = 0.10), and significant goodness-of-fit (Q = 231.87; df = 24; *p* < 0.001). Meta-regression confirmed higher incidence of IS in the Scandinavian region compared to that of the world studies (OR = 1.88; 95% CI 1.30–2.73; *p* < 0.001), and other 10 European studies (OR = 1.49; 95% CI 1.17–1.89; *p <* 0.001). The forest plots and summary incidence rates of sources above and below the 56^o^N latitudinal threshold were generated (Fig. 7). Additional sensitivity analyses excluding 2 birth cohort [47, 49] and 4 community-based studies [21, 24, 42, 49] did not alter these findings, or the global latitudinal effect (Fig. 8a and Fig. 8b).

The univariate meta-regression model of latitude and age-of-onset of IS, which was composed of 5 studies, determined that latitude did not predict age-of-onset (*p* = 0.38).

### Univariate meta-regression: Healthcare quality and Caucasian proportion

1 of 3 covariates representing healthcare quality was correlated to incidence. A positive relationship existed with physicians per capita (MD/capita) (OR = 1.57; 95% CI 1.09–2.27; *p* = 0.016). There was no effect of health expenditure per capita (*p* = 0.64), and health expenditure as percentage of GDP (*p* = 0.57).

No relationship existed between incidence and binary variable of national Caucasian proportion above 50% (*p* = 0.07).

### Univariate meta-regression: Time and GDP per capita

**Fig. 9:**
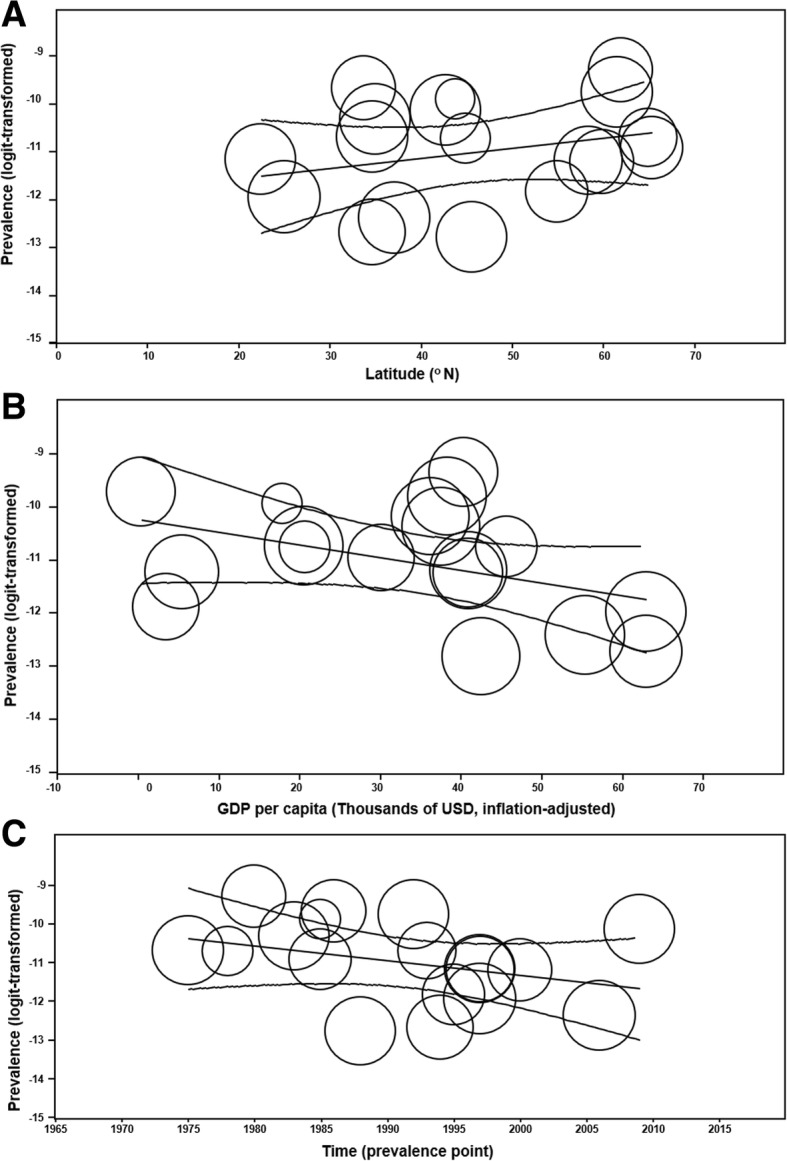
Scatter plots of prevalence. Prevalence of infantile spasms abstracted from 18 studies [15, 19, 30, 54–68] are represented as circles proportional to sample size and arranged in scatter plots according to various covariates. 95% confidence intervals and line of regression are shown. **a** Global prevalence is plotted against latitude. **b** Global prevalence is categorized by GDP per capita (inflation-adjusted to 2016 USD). **c** Global prevalence plotted against time, defined here as the prevalence point, which ranged from 1975 to 2009. Corresponding univariate meta-regression analysis demonstrated no effect of time on prevalence globally (*p* = 0.17)

Incidence was not predicted by time in the global (*p* = 0.07) (Fig. 8c), North American, (*p* = 0.66), or Asian (*p* = 0.88) contexts, although within Europe a negative correlation was observed (OR = 0.99; 95% CI 0.98–0.99; *p* = 0.0346). It was not predicted by GDP per capita globally (inflation-adjusted to 2016 USD, thousands) (*p* = 0.30). Conversely, the worldwide prevalence was negatively associated with GDP per capita (OR = 0.98; 95% CI 0.95–1.00; *p* = 0.047) (Fig. 9b) but not to time (*p* = 0.17) (Fig. 9c).

### Multiple meta-regression

Multiple meta-regression models consisting of latitude, time, and GDP per capita covariates were constructed. Continent was excluded because of its correlation with latitude and categorical status. There was positive association between latitude and incidence (OR = 1.02; 95% CI 1.01–1.03; *p* = 0.001) whereas GDP per capita (*p* = 0.54) and time (*p* = 0.39) were not associated. The multiple meta-regression model for prevalence revealed no associations.

The revised multiple meta-regression model incorporating MD/capita and the binary variable of Caucasian proportion demonstrated a positive relationship between incidence and only latitude (OR = 1.03; 95% CI 1.007–1.050; *p* = 0.01). The healthcare and ethnic covariates did not predict incidence in the presence of the other covariates (*p* = 0.76, *p* = 0.61 respectively).

### Analyses of sexual predisposition

From 14 incidence studies reporting sexual distribution, there was no significant predominance of male amongst patients (*p* = 0.056). The male proportion in the reference population was 49%. No association existed between the sample and population male proportions (*p* = 0.81). Amongst the 4 prevalence studies with sexual data, there was no majority of males (*p* = 0.14), and no association to the preference male proportion of 49% (*p* = 0.52).

### Analyses of publication Bias

The funnel plot of incidence studies with slight asymmetrical distribution was shown in Fig. 4b. The trim-and-fill method yielded an adjusted effect estimate of 0.25 cases/1000 LB, in comparison to the summary estimate of 0.249 cases/1000 LB. The funnel plot of prevalence studies was also shown in Fig. 5b and yielded an adjusted effect estimate of 0.010 cases/1000 population compared to the summary estimate of 0.015 cases/1000 population.

## Discussion

Clinical and animal studies have advanced our understanding of IS, yet there is persistent uncertainty of its pathophysiology and clinical variance [70]. These knowledge gaps suggest the existence of risk factors that influence the onset and activity of IS. The objective of the current study was to identify such factors with a novel, epidemiological approach.

The unique and specific clinical features described in the epileptic spasm seizure semiology, which historically and colloquially have been termed infantile spasms, have remained unchanged since their first description by Dr. William James West in 1841 [1]. Thus, by selecting studies explicitly reporting “infantile spasms”, “West Syndrome”, or its seizure type [2], we ensured that identification and inclusion of patients was complete irrespective of the date at which the study was conducted. An initial selection of 1015 articles was narrowed to 54 relevant studies on IS epidemiology. 39 publications of incidence determined a summary rate of 0.249 cases/1000 LB. This was within the previously-reported range of 0.2–0.5 cases/1000 LB. 18 publications provided a pooled prevalence of 0.015 cases/1000 population, which could not be compared to historical accounts due to unstandardized age ranges. The high I^2^ values in both databases indicated significant between-study heterogeneity and supported the notion that global IS epidemiology was not constant. Accordingly, a 3.3-Fold increase of summary incidence in Finland versus that of the U.S.A. was observed, in comparison to Cowan & Hudson’s seminal report of a 2.6-Fold difference in 1991 [6]. The basis of this inequality was unclear, and we hypothesized that it, and overall global heterogeneity, was the result of geographical factors. Therefore, we constructed univariate meta-regression analyses plotting incidence and prevalence against environmental covariates.

The continental meta-regression demonstrated increased incidence in Europe compared to that of Asia and North America. However, the comparable between-continent incidence after exclusion of Scandinavian studies indicated that this region featured populations of increased IS onset. Results of the meta-regression analyses of the latitudinal covariate were consistent with this pattern. We first determined the positive, global latitudinal gradient of IS incidence (Fig. 6a). The effect was unchanged by the exclusion of community-based and birth cohort studies into the predominantly hospital-based, cross-sectional database (Fig. 8a and Fig. 8b). Furthermore, latitude was the only covariate that maintained predictive value of incidence in the multivariate analyses. This was despite outlying studies, such as Lee & Ong, 2001 and Matsuo et al., 2001, that sampled equatorially-located populations to determine incidences of 0.31 cases/1000 LB which were higher than studies at comparable latitudes and the summary incidence. The latitudinal covariate could not predict all studies of the incidence database, which may be the result of factors not addressed in an epidemiological analysis and enhanced by the narrow study selection. Nonetheless, the latitudinal effect on global incidence, present in both univariate and multivariate analyses, was a novel finding that was further interrogated.

The sub-continental analyses of latitude and IS incidence failed to reproduce the trend in North American and Asia. This may partly be justified by the significant decline in study selection sizes of these regions, to 8 and 9 publications respectively. Conversely, the latitudinal effect was observed in the European analysis which consisted of 22 publications. The Scandinavian cohort exhibited particularly elevated incidence compared to the Equatorial selection. To explore the factors responsible for this difference, meta-analytic and regression models were constructed for that region separately. Forest plots visualized the relatively increased incidence and low heterogeneity of the Scandinavian selection (Fig. 7). The lack of latitudinal effect in this region was not unexpected considering the small range of event rate and geography. However, the absent latitudinal effect in the Equatorial cohort was consistent with the alternative hypothesis that the global latitudinal trend was elicited based on polar results.

The elevation of IS incidence in Scandinavia was traditionally attributed to regionally-specific superior healthcare and case detection. We did not identify literature attesting to a superior case detection system in the region. Furthermore, we conducted univariate analyses comparing global incidence with three national health system covariates determined from the World Health Organization Health Repository. An important consideration and potential limitation was of temporal incongruency: while study observation periods ranged from 1957 to 2014, the healthcare metrics were accessible only for 2014 to 2016. There is uncertainty that, based on publicly available data, the contemporary statistics reflect their historical counterparts. The assumption of superior Scandinavian healthcare is grossly consistent with 2014 to 2016 statistics demonstrating higher number of medical doctors per capita, ranked as 2, 4, 5, 9 for Sweden, Iceland, Denmark, and Finland respectively out of 17 countries. Nonetheless, we interpreted the univariate analyses with caution. We determined only one healthcare metric to be positively associated with incidence, the number of medical doctors per capita. However, in the multivariate meta-regression model, this covariate became non-significant. This indicated that if a superior Scandinavian detection system had existed, it did not affect reported incidence. This was supported by the methodological assessment that showed comparable ascertainment quality with Equatorial studies. Ultimately, we could provide little evidence of healthcare difference in Scandinavia contributing to elevated incidence of IS.

Whether the increased Scandinavian incidence is attributable to ethnicity remains unknown. Our attempt to elucidate an ethnic effect with the analysis of proportion of Caucasian individuals on incidence was inconclusive. However, this method had low sensitivity due to the small selection of countries and unstandardized data. This ethnic role is plausible based on its relationship to epilepsy prevalence as well as distinct patterns of disease epidemiology comparing Scandinavian populations internationally and internally [71–73]. Clarification of the effect is confounded by the hypothesized latitudinal effect; it may be necessary in future studies to examine a geographically limited, heterogenous selection of patients with ethnic stratification to identify a between-group effect. Furthermore, comparison of IS incidence between Scandinavian, or other at-risk ethnicities, to that of local populations across multiple nations and latitudes may demonstrate consistent elevation and reinforce suspicion of ethnic susceptibility. Ultimately, interrogation of the ethnic variable is highly dependent on the availability of data, and therefore continued accumulation of the epidemiologic database and strategic and creative design will likely be necessary to support these studies.

A further consideration in the determination of ethnic effect on IS incidence is the highly heterogenous determination of the seizure type: etiology which is proven in 60% of patients consists of dozens of pre- to post-natal disorders. Ethnicity may indirectly influence onset by affecting the incidence and/or predilection for seizure generation of these etiologies. Specifically, genetic disorders including chromosomal abnormalities and tuberous sclerosis complex constitute 15% of proven etiologies with unclear ethnic relationship [74]. We expect future studies to compile the currently-lacking ethnic data and corresponding etiologic distributions, to reveal nuanced patterns of etiological incidence, IS onset, and ethnicity.

The lack of latitudinal effect in the non-Scandinavian, Equatorial selection prevents confirmation of the latitudinal risk factor in IS onset. However, the decline in selection size from 39 to 26 sources represents a notable, potentially significant limitation especially in the context of known outlying studies in East-Asia. Furthermore, the relatively low probability value associated with this analysis (*p* = 0.10) indicated that the trend may be obscured. Dismissal of the latitudinal phenomenon would be premature on account of these statistical considerations. The existence of geo-epidemiological precedents in various immune-related disorders [75], viral infections [76], and the adaptive evolution of ACTN3 [77] indirectly support a latitudinal factor to IS incidence although only additional epidemiological studies on this topic may be conclusive. We expect that augmentation of the study selection outside of Scandinavia, including lacking southern hemispheric input, will reveal the Equatorial, and other regional, latitudinal trends.

If indeed the latitudinal effect exists, ultraviolet radiation (UVR), which is received in an inverse latitudinal manner, may be the relevant environmental agent. Environmental-neurophysiological interfaces have already been established in mice with seasonally-related, light-induced, mTOR and BDNF signalling-related dendritic morphologic adaptation [78], while UVR’s neurological role is thought to involve at least sleep duration [79], clock gene diversity [80], and development of multiple sclerosis [81]. In the case of IS, the UVR-epileptic interaction may be mediated by Vitamin D3, the secosteroid synthesized cutaneously in an UVR-dependent fashion, although infants primarily derive it from maternal contribution [82]. D3 depletion, which is associated to dysregulation of neurological immune activity [83] and neuronal hyper-excitability [84], in infants due to diminished maternal contribution secondary to latitude-related decrease in UVR exposure, may enable neurophysiological instability and seizures. This hypothesis is consistent with our analysis of age-of-onset of IS, which determined no association to latitude. Therefore, UVR would not induce seizure onset, but lower the seizure-threshold to predispose spasm generation by the underlying etiology.

A complementary environmentally-driven mechanism of IS development may involve melatonin, the key pineal hormone with modulatory effects on the circadian rhythm. Its secretion is physiologically suppressed by light exposure which results in a diurnal pattern of release, with peaking during night-time sleep. The expression of melatonin receptors across the brain suggest widespread and diverse neurological functions, and potential pathological roles, that remain poorly understood [85]. Evidence already exists for a proconvulsant effect of melatonin based on correlative studies of seizure frequency and elevated secretion during sleep and the pre-menstrual period [86]. Subsequent clinical studies have been inconclusive, however it is conceivable that the daily periods of prolonged darkness during winter nights in polar regions enhance the proconvulsant effect. Confirming the mechanisms of melatonin and D3-based processes of IS epileptogenesis is beyond the capability of the current database although elaboration of the latitudinal effect by etiology in future meta-analyses will be insightful.

Meta-regression of the other covariates was largely non-significant. Time was not a global predictor of incidence or prevalence (Fig. 8c and Fig. 9c). This was despite increasing survival of at-risk neonates and dissemination of IS awareness and diagnostic criteria, theoretically leading to increased incidence and prevalence [87]. Furthermore, the negative European correlation existing between time and incidence may have reflected increasingly effective management of etiological conditions across an expansive temporal scope. Perhaps these competing phenomena have manifested as overall static epidemiology, although there is no known study of this subject. GDP per capita was also negatively associated to IS prevalence globally, which suggested that national wealth modulated IS activity. This is a cautious conclusion considering that GDP was a non-specific measure of health care utilization. Finally, examination of the sex ratio of IS patients determined no correlation between proportion of study and population males. IS epidemiology has historically remarked a male preponderance [6] and this finding maintains the hypothesis that male sex is a risk factor. The relevant mechanisms may be indirect - as consequences of etiologies with poor outcomes more prevalent amongst males, and/or direct - as manifestations of the fundamental between-sex differences in brain morphology.

The main limitation of the current study was the restricted size of the database. The 54 studies questionably powered sub-analyses. Furthermore, recent human migration patterns could not be compiled due to lack of reporting, the foremost detractor of data validity. The methodological assessments also identified deficiency of unbiased case ascertainment in the incidence and prevalence databases, present at only 62 and 32% respectively. Adequate sample size was achieved in 46 and 16%, which likely enhanced the impacts of inadequate study selection. Fortunately, the other methodological domains were consistently fulfilled, and the overall scores and negligible publication bias indicated high quality of both databases.

This study has provided the first epidemiological evidence of a true geographical difference in IS incidence, which elaborates on the observations of Cowan & Hudson in 1991. This is apparent in the significant variability within the expanded global database, as well as between-continent and -region, that is independent of healthcare quality. The basis of the geographical effect is unconfirmed, although our subsequent analyses suggest a role of latitude and ethnicity, particularly in the Scandinavian region. Therefore, our findings justify the establishment of the global registry of IS epidemiology to facilitate detailed examination of this effect, and role of other environmental factors on IS generation, with additional studies and particularly Southern hemispheric input. Even with a limited database, our results have enabled and supported the hypotheses of Vitamin D3 and melatonin bases to seizure susceptibility, mechanisms which predispose IS onset independent of underlying etiology. These represent significant, emerging dimensions to IS pathophysiology. We anticipate that continued accumulation and analyses of epidemiological studies will expand this knowledge and clarify research targets. Specifically, future steps will likely interrogate the yet unconfirmed role of ethnicity, as demonstrated in other epilepsy syndromes, and incorporate novel concepts of the chronobiology of the mTOR pathway [78] and epigenetic factors in IS generation [84]. Consolidation of these clinical and pathophysiological findings may ultimately identify risk factors, motivate special scrutiny of susceptible populations, and support earlier identification and treatment to improve acute and chronic outcomes associated with IS [5, 88–91].

## Conclusions

Results from this systematic review and meta-analysis of the epidemiology of infantile spasms indicate an environmental dimension to the seizure type. Specifically, the significant heterogeneity of incidence and prevalence suggest a real geographical difference. The global latitudinal trend and exceptional Scandinavian incidence are consistent with the roles of a latitudinally-dependent risk factor or ethnicity respectively. However, further interrogation is difficult based on the sparsity and heterogeneity of available studies.

To expand on these novel findings and concepts, we advocate for the establishment of a global registry of infantile spasms incidence and prevalence. Such a global repository may guide the standardization of future original epidemiological studies and collect currently-lacking Southern hemispheric perspective, to facilitate the next generations of synthesis studies and ultimately inform areas of research and clinical intervention.

## Additional files

Additional file 1: Dataset of incidence studies. Table of the 39 incidence studies identified by author and publication year, as well as various covariates including time, location, Gross Domestic Product (GDP) per capita, sexual distribution, age-of-onset, and incidence and related demographic figures. USD: United States Dollars. AS: Asia. EUR: Europe. NA: North America. (XLSX 16 kb)

Additional file 2: Dataset of prevalence studies. Table of the 18 prevalence studies, identified by author and publication year, as well as various covariates including time, location, Gross Domestic Product (GDP) per capita, sexual distribution, age-of-onset, and prevalence and related demographic figures. USD: United States Dollars. AS: Asia. EUR: Europe. NA: North America. (XLSX 20 kb)

Additional file 3: Dataset of healthcare metrics and ethnic distribution by country for incidence database. Table of the 17 countries with incidence data, with accompanying summary adjusted prevalence figures, average latitudes, and national healthcare metrics. MD: medical doctor. CHE: Current Health Expenditure. (XLSX 15 kb)

## Abbreviations

GDP: Gross Domestic Product
ILAE: International League Against Epilepsy
IS: infantile spasms
LB: Live births
OR: Odds Ratio
USD: United States Dollars
UVR: Ultraviolet radiation
WS: West Syndrome
